# Common Genetic Factors Influence Hand Strength, Processing Speed, and Working Memory

**DOI:** 10.2188/jea.JE20130070

**Published:** 2014-01-05

**Authors:** Soshiro Ogata, Kenji Kato, Chika Honda, Kazuo Hayakawa

**Affiliations:** 1 Department of Health Promotion Science, Osaka University Graduate School of Medicine, Suita, Osaka, Japan; 1 大阪大学大学院医学系研究科保健学専攻総合ヘルスプロモーション科学講座; 2 Center for Twin Research, Osaka University Graduate School of Medicine, Suita, Osaka, Japan; 2 大阪大学大学院医学系研究科附属ツインリサーチセンター; 3 Division of Health Sciences, Kobe City College of Nursing, Kobe, Japan; 3 神戸市看護大学看護学部専門基礎科学領域健康科学分野

**Keywords:** hand strength, processing speed, working memory, twin study, cognitive decline

## Abstract

**Background:**

It is important to detect cognitive decline at an early stage, especially before onset of mild cognitive impairment and dementia. Processing speed and working memory are aspects of cognitive function that are associated with cognitive decline. Hand strength is an inexpensive, easily measurable indicator of cognitive decline. However, associations between hand strength, processing speed, and working memory have not been studied. In addition, the genetic and environmental structure of the association between hand strength and cognitive decline is unclear. We investigated phenotypic associations between hand strength, processing speed, and working memory and examined the genetic and environmental structure of the associations between phenotypes.

**Methods:**

Hand strength, processing speed (digit symbol performance), and working memory (digit span performance) were examined in monozygotic and dizygotic twin pairs. Generalized estimating equations were used to identify phenotypic associations, and structural equation modeling was used to investigate the genetic and environmental structure of the association.

**Results:**

Generalized estimating equations showed that hand strength was phenotypically associated with digit symbol performance but not with digit span performance. Structural equation modeling showed that common genetic factors influenced hand strength and digit symbol and digit span performance.

**Conclusions:**

There was a phenotypic association between hand strength and processing speed. In addition, some genetic factors were common to hand strength, processing speed, and working memory.

## Abstract (ja)

**背景:**

初期段階、特に軽度認知症および認知症発症前での認知機能低下を発見することは重要である。認知機能の処理速度とワーキングメモリーは認知機能低下に影響すると報告されている。また握力は簡易に測定でき安価な認知機能低下の指標になると報告されている。しかしながら握力と処理速度とワーキングメモリーの関連は不明である。また握力と認知機能の関連における遺伝環境構造は不明である。そのため本研究の目的は、握力と処理速度及びワーキングメモリーに関連があるか検討すること、加えてこれら変数間の関連における遺伝環境構造を検討することとする。

**方法:**

一卵性双生児ペアと二卵性双生児ペアの握力、処理速度(符号問題で測定)、及びワーキングメモリー(数唱で測定) を測定した。一般化推定方程式を用いて握力と処理速度及びワーキングメモリーの関連を分析した。構造方程式モデリングを用いて、これら変数間の関連における遺伝環境構造を検討した。

**結果:**

一般化推定方程式の結果、握力は符号問題と関連はあったが数唱とは関連がなかった。構造方程式モデリングの結果、握力と符号問題及び数唱に共通する遺伝要因が示された。

**結論:**

本研究により握力は処理速度と関連があることが示され、握力は初期段階の認知機能低下発見の指標となると示唆された。握力と処理速度及びワーキングメモリーに共通する遺伝要因が発見された。

## INTRODUCTION

Processing speed and working memory are aspects of cognition and influence cognitive decline.^1^^–^^5^ Hand strength is an inexpensive, easily measurable indicator of cognitive decline, such as dementia^6^^–^^12^; however, few studies have investigated associations between hand strength, processing speed, and working memory^12^ or assessed the genetic and environmental structures of the associations between hand strength and cognitive decline, including cognitive impairment, Alzheimer disease, and impaired processing speed and working memory.^6^^–^^12^ It is important to determine if hand strength is associated with processing speed and working memory because knowledge of such a relation might help to identify early cognitive decline—before onset of mild cognitive impairment (MCI) and dementia—which could help prevent dementia, reduce health care costs, and maintain quality of life and independence, as some people with cognitive decline gradually progress to mild cognitive impairment and Alzheimer disease.^13^^–^^17^ Additionally, it is worth investigating the genetic and environmental structure of the association between hand strength, processing speed, and working memory because knowledge of this structure could lead to identification of more-specific genetic and environmental factors and thus more-efficient screening methods and prevention strategies for MCI and dementia.

Processing speed is an index of the speed of central nervous system processing.^18^ Working memory represents the short-term activation, storage, and manipulation of information.^19^ Previous studies have shown that processing speed and working memory are cognitive functions associated with MCI and dementia^1^^–^^3^ and are useful for screening of MCI and dementia.^4^^,^^5^ Additionally, cross-sectional^6^^,^^7^ and longitudinal^8^^–^^12^ studies have shown an association between hand strength and cognitive decline, eg, cognitive impairment and dementia. Therefore, if hand strength is associated with processing speed and working memory, it might be useful in detecting cognitive decline before onset of MCI and dementia.

Twin studies are an excellent method of identifying genetic and environmental structures of associations between multiple observed variables. Twin studies can estimate the extent of genetic and environmental influences on associations between multiple observed variables by comparing similarities in monozygotic twins with those in dizygotic twins, based on the assumption that monozygotic twins share all their genes and dizygotic twins share, on average, only half their additive genes.^20^ If genetic influence is strong, similarities in monozygotic twins tend to be greater than those in dizygotic twins. However, when environmental influences are strong, the similarities in monozygotic twins tend to be the same as those in dizygotic twins, or the similarities in both monozygotic and dizygotic twins tend to be lower. Identifying the genetic and environmental structure of associations between hand strength, processing speed, and working memory could justify a search for more-specific genetic and environmental factors, which may lead to more-efficient screening methods and prevention strategies for MCI and dementia.

We tested the hypothesis that hand strength, processing speed, and working memory are associated. If this hypothesis proved to be correct, our second aim was to determine the genetic and environmental structure of these associations.

## METHODS

### Participants

This study used data collected in the Osaka University Aged Twin Registry in Japan. In this registry, twin pairs were recruited using several methods, such as newspaper advertisements, posters in hospitals, and referrals from midwives. The twins volunteered to participate in a cross-sectional comprehensive medical examination performed between 1984 and 1994. Zygosity was established on the basis of the results of the phenylthiocarbamide test and the 9 blood group systems, namely, ABO, RH (C, c, D, E, e), MN (M, N), Lewis (Le^a^, Le^b^), P (P_1_), Duffy (Fy^a^, Fy^b^), Kidd (JK^a^, JK^b^), Kell (K), and Diego (Di^a^). A twin pair was classified as monozygotic if all blood types were consistent within a twin pair and as dizygotic if any blood type result was not consistent within a twin pair. This classification methodology is known to be 95% accurate in determining twin zygosity.^21^^,^^22^ This study was approved by the Osaka University Ethical Review Committee.

In the examination, 539 participants were recruited. In this study, twins aged between 30 and 75 years were evaluated because processing speed is thought to begin declining at age 30 years and because the Japanese government requests that all Japanese people aged between 40 and 75 years undergo a health examination. Participants with unknown zygosity and opposite-sex dizygotic twins were excluded. Ultimately, we analyzed 348 of the 539 participants: 133 monozygotic twin pairs (*n* = 266) and 41 same-sex dizygotic twin pairs (*n* = 82). In Table 1, the χ^2^ test, T test, and F test showed that differences in proportions, means, and variances (including sex and age) were not significant between monozygotic and dizygotic twin pairs (all *P* values > 0.05).

**Table 1.** Descriptive statistics

Variables	MZ *n* = 266 (133 pairs)	DZ *n* = 82 (41 pairs)	% of missing values	2-sided *P* values	
	*n* (%)		%	χ^2^ test	

Males	164 (61.65)	52 (63.41)	0	0.88	

	Mean (SD)			T test	F test

Age, years	56.3 (8.86)	55.24 (10.43)	0	0.36	0.06
Height, cm	156.8 (7.54)	158.68 (8.31)	0	0.06	0.26
Weight, kg	55.53 (8.65)	57.05 (9.57)	0	0.17	0.24
Right hand strength, kg	35.29 (9.34)	34.78 (9.67)	4.31	0.68	0.68
Left hand strength, kg	33.52 (9.48)	33.71 (9.4)	4.60	0.88	0.95
Hand strength score, kg	34.4 (9.18)	34.24 (9.31)	4.60	0.90	0.85
Digit symbol score	48.36 (12.9)	45.11 (15.13)	7.18	0.07	0.06
Digit span forward score	6.22 (1.15)	6.26 (1.35)	5.17	0.79	0.08
Digit span backward score	4.31 (1.13)	4.11 (0.94)	5.46	0.18	0.06
Digit span total score	10.53 (1.96)	10.36 (1.9)	5.46	0.51	0.76

Abbreviations: MZ, monozygotic; DZ, dizygotic.

### Measurements

#### Hand strength

A trained interviewer measured hand strength (in kg) using a Smedley hand dynamometer (No. 92720, MIS, Tokyo). The minimum unit of measurement was 0.5 kg. Grip size was adjusted so that each participant felt comfortable while squeezing the apparatus. The participant was instructed and verbally encouraged to squeeze the hand dynamometer as hard as possible. One trial each was performed for the right and left hands, and the right hand was measured first. The strength of the stronger side (ie, right or left) was used to determine hand strength score, which was used in the analyses. A higher hand strength score indicated greater hand strength.

#### Processing speed

To measure processing speed, a trained interviewer conducted the digit symbol substitution test, contained in the Wechsler Adult Intelligence Scale, First Edition.^23^^,^^24^ The participant was given a sheet with 9 different digit-symbol pairs and was asked to refer to a key on the sheet to pair random digits with their matching symbols as quickly as possible within 90 seconds. The digit symbol score was calculated based on the total number of correct digit-symbol matches and ranged from 0, worst performance, to 90, best performance.

#### Working memory

To measure working memory, a trained interviewer conducted the digit span test, contained in the Wechsler Adult Intelligence Scale, First Edition.^23^ The test consisted of digits-forward and digits-backward conditions. In the digits-forward condition, the interviewer read aloud a series of numbers of increasing length, and the participant was instructed to repeat the numbers in the same order. In the digits-backward condition, the interviewer read aloud a series of numbers of increasing length, but the participant was instructed to repeat the numbers in reverse order. The sequences were increased in length by 1 unit in each subsequent trial until the participant failed 2 trials in a row of the same sequence length. The digits-forward condition consisted of sequences of 3 to 9 units, and the digits-backward condition consisted of sequences of 2 to 8 units. Forward and backward scores were based on the longest length of correct answers in each condition. A total score then was obtained by adding the forward and backward scores (range, 0–17). A higher digit span score indicated better working memory performance.

### Statistical analyses

#### Phenotypic associations between hand strength, processing speed, and working memory

To investigate associations between hand strength, processing speed, and working memory, we used generalized estimating equations (GEE), which control for clustering of twins within a pair. Using GEE, we obtained standardized coefficients and 95% CIs adjusted for age and sex with an exchangeable working correlation matrix. We used multiple imputation to adjust for missing data only when performing GEE.^25^^–^^27^ We used the gee 4.13-18 package for gee analyses, and the mice 2.13 package for multiple imputation, in R statistical software, version 2.15.2.^28^

#### Structural equation modeling to decompose covariance into genetic and environmental components

To assess the genetic and environmental structure of phenotypic covariance between hand strength, processing speed, and working memory, we used structural equation modeling for multivariate genetic analyses. Based on the assumption that monozygotic twins share all their genes and dizygotic twins share on average only half their additive genes, multivariate genetic analyses decompose similarities measured by the covariance matrix of the 3 phenotypic variables among respective monozygotic and dizygotic twin pairs into the following 3 of 4 latent variables: (1) additive genetic influences (A), (2) nonadditive genetic influences (D) or shared environmental influences (C), and (3) nonshared environmental influences (E).^20^ The A effect is assumed to be the sum of polygenes whose effects are small and additive to form a quantitative phenotype. The D effect is assumed to be an interaction between alleles at the same locus (ie, dominance) or different loci (ie, epistasis). The C effect makes family members alike not from heredity but from the common environment shared by family members. The E effect makes family members different even if they live together.

**Figure 1.**
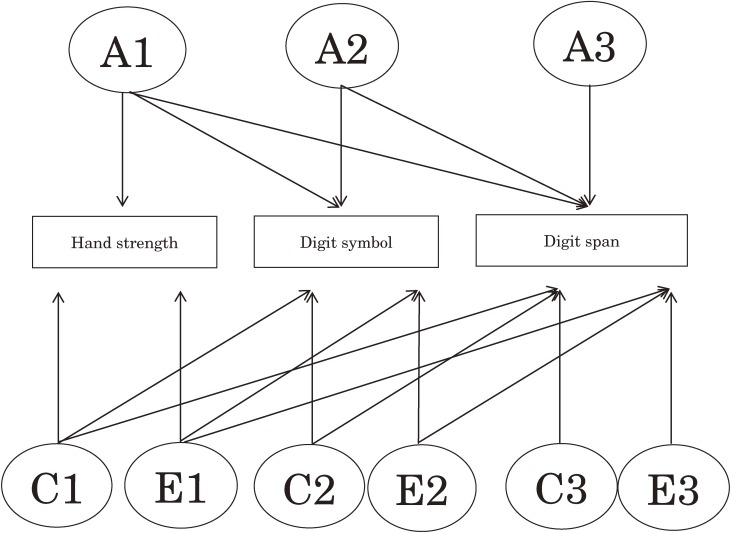
Full ACE Cholesky model. Rectangles represent observed variables, ellipses represent latent variables, and arrows indicate a directional effect. Abbreviations: A, additive genetic; C, shared environment; E, nonshared environment.

We used Cholesky decomposition models, which are 1 of the multivariate genetic analyses, to decompose covariance between observed variables (phenotypes) into latent variables (genetic and environmental effects).^20^ The Cholesky decomposition model includes equal numbers of latent variables (eg, A1, A2, A3, C1, C2, C3, E1, E2, and E3 in this study) and observed variables (eg, hand strength, digit symbol performance, and digit span performance in this study) for each independent source of variance (ie, A, C, and E). The first latent variables load on all phenotypes, the second load on all but the first, and the third on all but the first and second (Figure 1).

An aim of genetic analyses is to find the best-fitting model that is most theoretically acceptable and parsimonious. The best-fitting model was selected on the basis of the likelihood ratio test and Akaike information criterion. To identify the best-fitting model, we performed model fitting in 2 steps. Because C and D effects cannot be estimated in the same model, we first compared the full ACE and ADE Cholesky models with the fully saturated model. The fully saturated model implies that covariance is treated as a free parameter, which will be equal to the sample covariance. Through these comparisons, we determined which of the full ACE or ADE Cholesky models was plausible. We then constructed submodels from the full Cholesky model by dropping parameters and latent variables and then comparing these submodels with the full Cholesky model. In structural equation modeling, we used a regression technique to obtain standardized path coefficients with 95% CIs adjusted for age and sex.^29^ In structural equation modeling, we used the full information maximum likelihood method to adjust for missing data.^30^ The genetic analyses were performed using the OpenMx 1.3 package^31^ in R statistical software version 2.15.2.^28^

## RESULTS

Table 1 shows the descriptive statistics. The χ^2^, T, and F tests showed that differences in proportions, means, and variances between monozygotic and dizygotic twin pairs were not significant for all variables (*P* > 0.05 for all tests).

Tables 2 and 3 show the standardized partial regression coefficients in GEE analyses. These GEE analyses can be considered identical to analyses of an unselected non-twin population. After adjusting for age and sex, standardized partial regression coefficients indicated that hand strength was significantly associated with digit symbol performance but not digit span performance. Therefore, hand strength was phenotypically associated with digit symbol performance but not with digit span performance. We obtained similar results in complete-case analysis, a method of handling missing data.

**Table 2.** Standardized partial regression coefficients for digit symbol performance in GEE analysis

Explanatory variable	Coefficient	Lower limit of 95% CI	Upper limit of 95% CI
Multiple imputation used to adjust for missing data			

Sex (female)	0.11	−0.02	0.25
Age	−0.55	−0.67	−0.43
Hand strength	0.18	0.06	0.31

Complete-case analysis used to adjust for missing data			

Sex (female)	0.11	−0.01	0.24
Age	−0.67	−0.82	−0.52
Hand strength	0.18	0.06	0.30

Abbreviations: GEE, generalized estimating equations.

**Table 3.** Standardized partial regression coefficients for digit span performance in GEE analysis

Explanatory variable	Coefficient	Lower limit of 95% CI	Upper limit of 95% CI
Multiple imputation used to adjust for missing data			

Sex (female)	−0.05	−0.22	0.12
Age	−0.17	−0.32	−0.02
Hand strength	−0.04	−0.21	0.13

Complete-case analysis used to adjust for missing data			

Sex (female)	−0.03	−0.20	0.13
Age	−0.20	−0.40	−0.01
Hand strength	−0.03	−0.20	0.15

Abbreviations: GEE, generalized estimating equations.

**Figure 2.**
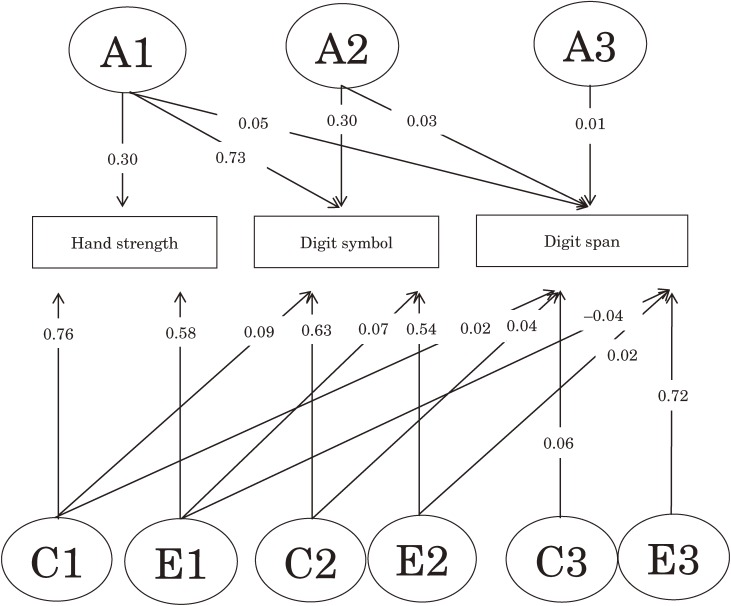
Standardized path coefficients for hand strength, digit symbol performance, and digit span performance in the full ACE Cholesky model. Rectangles represent observed variables, ellipses represent latent variables, and arrows indicate a directional effect. Abbreviations: A, additive genetic; C, shared environment; E, nonshared environment.

**Figure 3.**
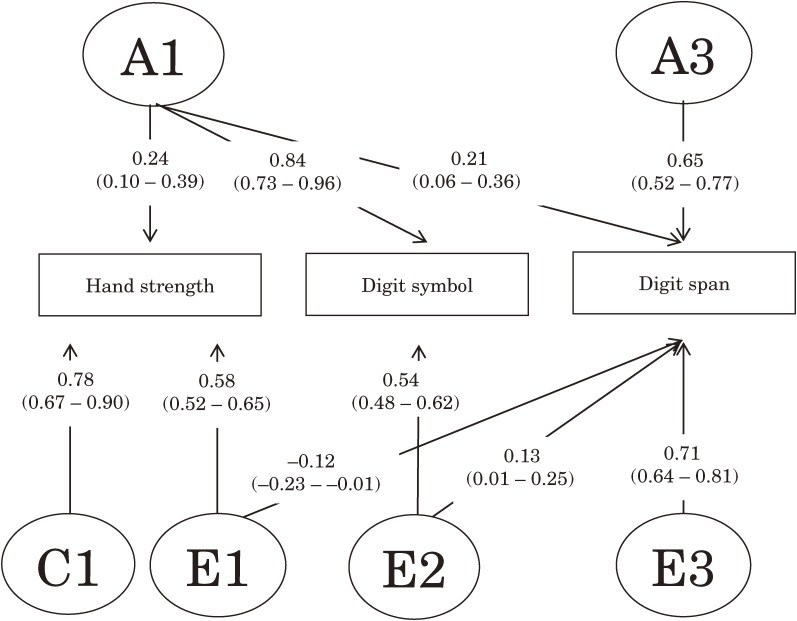
Standardized path coefficients and 95% CIs for hand strength, digit symbol performance, and digit span performance in the best-fitting model. Rectangles represent observed variables, ellipses represent latent variables, and arrows indicate a directional effect. Abbreviations: A, additive genetic; C, shared environment; E, nonshared environment.

Table 4 shows the results of model fitting. When the full ACE and ADE Cholesky models were compared with the fully saturated model, the Akaike information criterion value of the full ACE Cholesky model was the lowest of the 3 full models. According to the likelihood ratio test, the full ACE Cholesky model fitted the data well (Δ −2 log likelihood = 23.70, Δ degrees of freedom = 33, *P* = 0.88). Figure 2 shows the standardized path coefficients of the full ACE Cholesky model. After step-by-step dropping of nonsignificant paths in the full ACE Cholesky model, model 11 was the best fitting model, based on having the lowest Akaike information criterion value (Δ −2 log likelihood = 5.48, Δ degrees of freedom = 8, *P* = 0.70). Figure 3 shows the best-fitting model, which comprised 2 genetic and 4 environmental factors. Common genetic factors (A1) influenced hand strength, digit symbol performance, and digit span performance, whereas unique genetic factors (A3) influenced only digit span performance. Common nonshared environmental factors (E1) influenced hand strength and digit span performance, whereas common nonshared environmental factors (E2) influenced digit symbol performance and digit span performance. Unique nonshared environmental factors (E3) influenced only digit span performance, whereas unique shared environmental factors (C1) influenced only hand strength. Estimates of heritability of hand strength, digit symbol performance, and digit span performance were 6%, 71%, and 47%, respectively. In complete-case analysis, model 11 was again the best fitting model, and all paths except those from E1 and E2 to digit span performance were significant.

**Table 4.** Results of model fitting

Models	AIC
1) Fully saturated model	3856.90
2) Full ADE Cholesky model	3826.39
3) Full ACE Cholesky model	3814.59
4) Same as model 3, but drop a path from C3 to digit span	3813.78
5) Same as model 4, but drop a path from C2 to digit span	3812.42
6) Same as model 5, but drop a path from C2 to digit symbol	3812.31
7) Same as model 6, but drop a path from C1 to digit span	3810.34
8) Same as model 7, but drop a path from C1 to digit symbol	3808.42
9) Same as model 8, but drop a path from E1 to digit symbol	3807.20
10) Same as model 9, but drop a path from A2 to digit span	3805.39
11) Same as model 10, but drop a path from A2 to digit symbol	3804.08
12) Same as model 11, but drop a path from A3 to digit span	3832.82
13) Same as model 11, but drop a path from A1 to digit span	3810.03
14) Same as model 11, but drop a path from A1 to digit symbol	3900.97
15) Same as model 11, but drop a path from A1 to hand strength	3812.75

Abbreviation: AIC, Akaike information criterion; A, additive genetic; D, nonadditive genetic; C, shared environmental; E, nonshared environmental.

## DISCUSSION

Using a genetically informative sample of twins, we investigated associations between hand strength, processing speed, and working memory as well as the genetic and environmental structure of the associations between these variables. Hand strength was associated with processing speed (ie, digit symbol performance). In addition, we identified genetic factors common to hand strength, processing speed, and working memory (ie, digit span performance).

We found that greater hand strength was significantly associated with higher processing speed. Results of GEE analysis showed that greater hand strength was significantly associated with better digit symbol performance. Previous studies have reported an association between hand strength and cognitive decline.^6^^–^^12^ Because processing speed is an important marker of cognitive decline,^2^^,^^4^^,^^5^ previous results showing an association between hand strength and cognitive decline are congruent with the present results. However, the direction of the causal relationship between hand strength and processing speed remains unclear for the following reasons. First, a longitudinal study found that a decline in processing speed precedes the onset of muscle weakness as reflected by decreased hand strength.^12^ Second, our study had a cross-sectional design. However, measuring hand strength is easy and inexpensive; thus, we believe it can be used as an indicator of processing speed.

We also found genetic factors common to hand strength, processing speed, and working memory. Results of structural equation modeling indicate that common genetic factors (A1) significantly influenced hand strength, digit symbol performance, and digit span performance. Previous findings suggest that the mechanisms of the association between muscle strength (measured by hand strength) and cognitive function have a neurobiologic basis, which affects both cognitive and motor functional decline.^9^^,^^11^^,^^12^ In addition, a study reported genetic factors common to a working memory measure, a processing speed measure, and P300 latency.^32^ P300 is an event-related potential component—that is, the measured brain response directly resulting from a specific sensory, cognitive, or motor event^33^—and is used to measure processing speed. A previous study reported that physically active individuals have faster P300 latency than do sedentary individuals.^34^ Hand strength is used to measure physical capability.^35^ Therefore, it is possible that A1 indicates genetic influence on neural activity reflecting P300 latency. Another possibility is that A1 reflects genetic influence on pathogenic factors common to muscle strength and cognitive functions, as studies have suggested that mechanisms of the association between muscle strength (measured by hand strength) and cognitive function may be common pathogenic factors in conditions such as vascular diseases, frailty, declines in levels of sex hormones, cytokine activation, high levels of inflammation markers, and oxidative stress.^9^^,^^11^

Although phenotypic analyses (GEE) showed an insignificant phenotypic association between hand strength and working memory, genetic analyses revealed a significant genetic and environmental association between these variables. The phenotypic association between hand strength and digit span performance disappeared because the positive genetic and negative environmental correlations between hand strength and digit span performance negated each other. In fact, a previous study reported that hand strength was significantly associated with working memory at the phenotypic level.^12^ The discrepancy between present and past results may be due to differences in sample size and participant age (*n* = 1043; 55–85 years).

A notable strength of our study is that it was based on a genetically informative sample, which allowed us to investigate the genetic and environmental structure of the phenotypic associations between traits. In addition, because the sample was relatively young (mean age, 56.05 [SD, 9.19] years), it likely covered a wide range of cognitive abilities.

This study had limitations. First, because of the cross-sectional design, the direction of the causal relationship remains unclear. However, as hand strength is easily and inexpensively measured, we believe it can be used as an indicator of processing speed. Second, although this study provided new evidence regarding the mechanisms of the association between hand strength and cognitive decline, we did not collect data from direct measurements of brain function. To clarify the mechanisms involved, we have begun using magnetoencephalography to collect data from twin pairs. This allows us to directly measure the event-related field, which is equivalent to event-related potential. Third, the sample size was smaller for dizygotic twin pairs than for monozygotic twin pairs. One reason of this disproportionality was that more monozygotic twin births than dizygotic twin births occurred in Japan between 1951 and 1990^36^; this period included the birth years of participants aged from 31–44 years. In addition, this disproportionality might have influenced the results of standardized path coefficients in the best-fitting model in structural equation modeling.

In conclusion, our findings revealed an association between hand strength and processing speed, as well as genetic factors common to hand strength, processing speed, and working memory. We believe that our results may assist in detecting cognitive decline at an early stage, before the onset of MCI and dementia, and in understanding the genetic and environmental structure of the association between hand strength and cognitive decline.

## ONLINE ONLY MATERIALS

Abstract in Japanese.
