# Supplementary material for: Common Genetic Factors Influence Hand Strength, Processing Speed, and Working Memory
Source: J Epidemiol. 2014 Jan 5;24(1):31–8. doi: 10.2188/jea.JE20130070 (PMC3872522; doi:10.2188/jea.JE20130070)
Supplement: Abstract in Japanese. [file je-24-031-s001.pdf]

握力、処理速度、およびワーキングメモリーに共通する遺伝要因

尾形宗士郎[1,2]、加藤憲司[3]、本多智佳[2]、早川和生[1,2]

[1]大阪大学大学院医学系研究科保健学専攻総合ヘルスプロモーション科学講座

[2]大阪大学大学院医学系研究科附属ツインリサーチセンター

[3]神戸市看護大学看護学部専門基礎科学領域健康科学分野

背景： 初期段階、特に軽度認知症および認知症発症前での認知機能低下を発見することは重要である。認知機能の処理速度とワーキングメモリーは認知機能低下に影響すると報告されている。また握力は簡易に測定でき安価な認知機能低下の指標になると報告されている。しかしながら握力と処理速度とワーキングメモリーの関連は不明である。また握力と認知機能の関連における遺伝環境構造は不明である。そのため本研究の目的は、握力と処理速度及びワーキングメモリーに関連があるか検討すること、加えてこれら変数間の関連における遺伝環境構造を検討することとする。

方法： 一卵性双生児ペアと二卵性双生児ペアの握力、処理速度（符号問題で測定）、及びワーキングメモリー（数唱で測定） を測定した。一般化推定方程式を用いて握力と処理速度及びワーキングメモリーの関連を分析した。構造方程式モデリングを用いて、これら変数間の関連における遺伝環境構造を検討した。

結果： 一般化推定方程式の結果、握力は符号問題と関連はあったが数唱とは関連がなかった。構造方程式モデリングの結果、握力と符号問題及び数唱に共通する遺伝要因が示された。

結論： 本研究により握力は処理速度と関連があることが示され、握力は初期段階の認知機能低下発見の指標となると示唆された。握力と処理速度及びワーキングメモリーに共通する遺伝要因が発見された。

キーワード： 握力、処理速度、ワーキングメモリー、双生児研究、認知機能低下
